# Tailored acoustic metamaterials. Part I. Thin- and thick-walled Helmholtz resonator arrays

**DOI:** 10.1098/rspa.2022.0124

**Published:** 2022-06

**Authors:** Michael J. A. Smith, I. David Abrahams

**Affiliations:** Department of Applied Mathematics and Theoretical Physics, University of Cambridge, Wilberforce Road, Cambridge CB3 0WA, UK

**Keywords:** acoustics, Helmholtz resonator, two-dimensional array, matched asymptotic expansions, multipole methods, metamaterial

## Abstract

We present a novel multipole formulation for computing the band structures of two-dimensional arrays of cylindrical Helmholtz resonators. This formulation is derived by combining existing multipole methods for arrays of ideal cylinders with the method of matched asymptotic expansions. We construct asymptotically close representations for the dispersion equations of the first band surface, correcting and extending an established lowest-order (isotropic) result in the literature for thin-walled resonator arrays. The descriptions we obtain for the first band are accurate over a relatively broad frequency and Bloch vector range and not simply in the long-wavelength and low-frequency regime, as is the case in many classical treatments. Crucially, we are able to capture features of the first band, such as low-frequency anisotropy, over a broad range of filling fractions, wall thicknesses and aperture angles. In addition to describing the first band we use our formulation to compute the first band gap for both thin- and thick-walled resonators, and find that thicker resonator walls correspond to both a narrowing of the first band gap and an increase in the central band gap frequency.

## 1. Introduction

In recent years, researchers within the metamaterials and composite materials communities have uncovered a vast array of media exhibiting interesting and unexpected wave scattering properties. These have ranged from ultralow frequency band gaps to one-way edge states and negative refraction [1–3], in a diverse range of wave settings, for example, from acoustics and elasticity through to electromagnetism. The ongoing development of novel materials remains a very topical and important endeavour for mathematicians, physicists, engineers and materials scientists alike. In order to describe compactly the performance of meta/composite materials, significant attention has been directed towards the efficient calculation of *band diagrams* and on obtaining *effective medium descriptions*, i.e. homogenization of the medium, in a range of settings.

From across the literature, a diverse selection of homogenization tools have likewise emerged, ranging from fully numerical procedures to analytical methods that yield elegant closed-form expressions [1,2,4]. One established and well-known analytical procedure combines multipole methods with conventional asymptotic methods to obtain closed-form descriptions for two-dimensional arrays of cylinders embedded in a background material [4,5]. These descriptions for non-resonant arrays of scatterers have proven exceptionally useful for developing highly tuned materials whose properties lie between those of the inclusion and matrix phase (analogously to the way an array of resistors combined in series or in parallel form effective resistances). However, as with the vast majority of effective medium descriptions, the analytical representations describe the first band surface only at both low frequencies and at long wavelengths. In place of this limited description, it is much more advantageous to obtain descriptions of the first band over a broader range.

In this work, we attempt to obtain simple asymptotic descriptions of the first band surface over the entire Brillouin zone for a two-dimensional Helmholtz resonator array, and more generally, present a multipole formulation for computing band diagrams over a wide frequency range. We use a combination of multipole methods [4,6] and the method of matched asymptotic expansions [7–9] to obtain results for an array of thin-walled resonators, deriving and providing a small correction to the result published in Llewellyn Smith & Davis [10], as well as extending treatments to obtain crucial next-order corrections that capture the anisotropy of the medium. We also derive an analogous formulation for thick-walled resonator arrays and present corresponding results. Our multipole solutions rely upon a careful choice of ansatz for the leading-order outer fields; these assume that a monopole contribution from the aperture, in addition to a cylindrical harmonic basis, is able to accurately describe the modal field at low frequencies. For a very narrow aperture this is a perfectly reasonable assumption, and we are able to show that a monopole contribution is generally able to recover the first band even for very wide aperture values. That said, our treatment may be extended to much higher frequencies by considering dipole, quadrupole or higher-pole source contributions at the aperture mouth.

The expressions obtained here for the first band will prove useful for practical applications, admitting closed-form expressions for both the phase and group velocity inside the crystal, for example. In addition to capturing Bloch vector and frequency dependence (spatial and frequency dispersion), our descriptions also give the width of the first (subharmonic) band gap in a range of resonator array configurations. To the best of our knowledge, we are unaware of such analytical results for two-dimensional resonant arrays, although there are close similarities to a lowest-order result for *thin-walled* Helmholtz resonator arrays [10]. That said, relatively few analytical studies of this nature exist due to the complexity involved in their derivation, although there is an extensive literature on numerical results (e.g. [11,12]). In this work, we do not rely upon *lumped-element models* or *lumped acoustic elements*, which have been used extensively in the literature to model Helmholtz resonators; such treatments replace the resonator with an equivalent mass-and-spring or circuit, which has proven useful in the past for describing resonators in the deeply long-wavelength regime [13].

The descriptions we obtain for the first band complements other work in the literature on two-dimensional arrays of resonators governed by Helmholtz’s equation, such as work on thick cylindrical resonators possessing multiple apertures [14,15] that exhibit effects such as negative refraction. Other Helmholtz equation studies of this type include work on two-dimensional arrays of thick-walled split-ring resonators [16] and two-dimensional arrays of closely packed solid cylinders [17]. Estimates for the upper and lower bounds of the first band gap in elastic resonator array problems have also been considered [18]. Research on resonator arrays has also been conducted extensively for Maxwell’s equations, including a numerical study on determining effective optical constants of a two-dimensional array of infinitesimally thin split-ring resonators [19]. Another related area examines arrays of gas bubbles in liquids; the fundamental frequency at which the bubble wall oscillates is analogous to a Helmholtz resonance and induces low-frequency band gaps within the fluid medium [20]. There has also been interest within the water waves community on arrays of graded thin-walled Helmholtz resonators, which can exhibit strong field amplification, a feature which may prove useful in energy harvesting systems [21].

The outline of this paper is as follows. First we present the boundary value problem for a two-dimensional doubly periodic array of thin-walled Helmholtz resonators in §2. We then set up the matching scheme by examining the field close to an aperture in §3, and derive field asymptotics as we move out from this inner region. Next, we construct an *outer solution* outline in §4, where the presence of the small aperture is modelled by a simple source term. We then conduct asymptotic matching in §4c to obtain our eigensystem in §4d. This allows us to derive the leading-order dispersion equation for the first spectral band in §4e followed by its first-order correction in §4f. In §5 we consider numerics for a selection of geometries to demonstrate the efficacy of our approximations. This is followed by a treatment for thick-walled resonators in §6, where we outline all modifications and present additional numerical results. Finally we offer some concluding remarks in §7.

## 2. Problem formulation

We consider a two-dimensional square array of thin-walled resonators spaced a distance $\bar{d}$ apart that are modelled as cylinders of radius $\bar{b}$, each containing an aperture of arc length $2\bar{\ell }$ centred about the central angle $\theta_{0}$. These are immersed in an acoustic medium of infinite extent satisfying the two-dimensional scalar Helmholtz equation

2.1 $$(\partial_{\bar{x}}^{2}+\partial_{\bar{y}}^{2})\bar{\phi }+k^{2}\bar{\phi }=0,$$

with Neumann boundary conditions imposed on all resonator walls, and where $\partial_{\bar{x}}$ denotes a partial derivative with respect to $\bar{x}$, for example. The overbar is used to denote dimensional quantities, and we take $\bar{\phi }$ to be the steady-state monochromatic field oscillating at angular frequency ω, i.e. the observed time-dependent field is $\text{Re}\{\bar{\phi }\exp(-\text{i}\omega t)\}$, but we omit reference to this henceforth for brevity. Owing to the symmetries of the full array problem, we consider Helmholtz’s equation in the fundamental unit cell $\bar{\Omega }$ containing a single resonator and satisfying Bloch conditions between adjacent cells (defined below). Here, $\left(\bar{x},\bar{y}\right)$ represents dimensional Cartesian coordinates, $k=\omega \sqrt{\rho /B}$ the wave number, ω the angular frequency, B the bulk modulus and ρ the mass density of the surrounding acoustic medium. For future reference, we also denote the dimensional Bloch vector by $\left({\bar{k}}_{Bx},{\bar{k}}_{By}\right)$, and we note that all Cartesian dimensional quantities possess an overbar, along with $\bar{\phi }(\bar{x},\bar{y})$, but that the remaining quantities do not (i.e. ρ, ω, B and k).

In order to reduce the number of parameters, and to better understand the mathematical treatment to follow, we non-dimensionalize as

**Figure 1.**
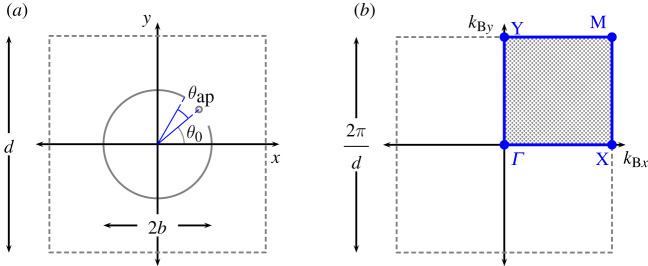
(*a*) Fundamental (dimensionless) unit cell for a square array of period d containing a thin cylindrical resonator of radius b with aperture of arc length 2ℓ centred at $\left(b,\theta_{0}\right)$ in polar coordinates, i.e. with half-angle subtended by the aperture given by $\theta_{\mathrm{ap}}=\ell /b$; (*b*) corresponding unit cell in reciprocal space, for high symmetry values of $\theta_{0}$, expressed in terms of non-dimensional Bloch coordinates $\left(k_{Bx},k_{By}\right)$ with irreducible Brillouin zone shaded, bounded by blue lines and marked with vertices Γ=(0,0), X=(π/d,0), Y=(0,π/d) and M=(π/d,π/d). (Online version in colour.)

2.2 $$\bar{x}=\frac{x}{k},\;\bar{y}=\frac{y}{k},\;\bar{b}=\frac{b}{k},\;\bar{d}=\frac{d}{k},\;\bar{\ell }=\frac{\ell }{k},\;\mathrm{and}\;({\bar{k}}_{Bx},{\bar{k}}_{By})=k(k_{Bx},k_{By}),$$

to obtain the governing equations for our problem inside the unit cell Ω, shown in figure 1, in the form

2.3a $$(\partial_{x}^{2}+\partial_{y}^{2}+1)\phi =0\;\text{for}\;(x,y)\in \Omega \setminus S,\;\text{with}\;$$

and

2.3b $$\partial_{r}\phi =0\;\text{for}\;(x,y)\in S\;\text{and}\;\phi (x+md,y+nd)=\phi (x,y)\;{\text{e}}^{\text{i}(k_{Bx}md+k_{By}nd)},$$

where $\bar{\phi }(\bar{x},\bar{y})=\phi (x,y)$ and we represent the infinitesimally thin cylinder with an aperture by S=(bcos⁡θ,bsin⁡θ) with $\theta \in (\theta_{0}+\theta_{\mathrm{ap}},2\pi -\theta_{\mathrm{ap}}+\theta_{0})$. The definition for S prescribes an infinitesimally thin resonator of radius b with an aperture centred at $\theta_{0}$ and a half-width angle of $\theta_{\mathrm{ap}}=\ell /b$ (i.e. a gap with total arc length $2\ell =2k\bar{\ell }$). For the Bloch condition (2.3*b*), we define the integers m,n∈Z and lattice period for a square array d. In this work, we treat half the arc length for the aperture as the small parameter ε=ℓ, as this is the appropriate regime for resonance, and we begin by considering the problem local to the aperture to commence our asymptotic solution.

## 3. Inner problem formulation

As outlined in the literature [7–9], solutions obtained using the method of matched asymptotic expansions require both an inner and an outer solution, in addition to a rigorous matching rule. In general, the inner solution describes the near field (i.e. close to a boundary or object), and the outer solution describes the behaviour in the far field (i.e. far away from the boundary or object) [7]. For our problem, the curvature of the resonator wall boundary is locally zero as we focus in on the aperture, and so, the walls may be regarded as flat (i.e. we take the asymptotic limit as the radius of the cylinder is long relative to the aperture size). This idea of vanishing local curvature is equivalent to the concept of a plane wave, which formally corresponds to a source point placed at infinity.

As a first step, we rotate and translate the array via $\left(\tilde{x},\tilde{y})\mapsto (x\sin\theta_{0}-y\cos\theta_{0},x\cos\theta_{0}+y\sin\theta_{0}-b\right)$ so that the aperture in the fundamental cell is centred about the origin. Subsequently, we introduce the inner scaling

3.1 $$X=\frac{\tilde{x}}{\varepsilon }\;\text{and}\;Y=\frac{\tilde{y}}{\varepsilon },$$

as well as posing the regular expansion $\phi =\sum_{m=0}^{\infty }\varepsilon^{m}\Phi_{m}(X,Y)$. Substituting the scaling (3.1) and expansion into the Helmholtz equation (2.3*a*) and Neumann condition (2.3*b*), we obtain the leading-order inner problem given by

**Figure 2.**
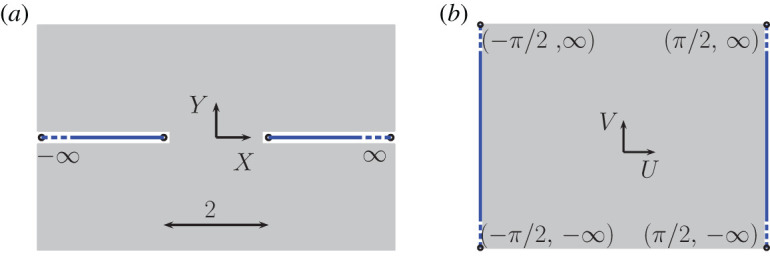
Inner problem domain comprising an infinitely extending screen with resonator mouth of length 2 in terms of the inner coordinates (X,Y); (*b*) equivalent representation obtained via the mapping W=arcsin⁡(Z) in terms of transformed inner coordinates (U,V). (Online version in colour.)

3.2 $$(\partial_{X}^{2}+\partial_{Y}^{2})\Phi =0\;\text{for }(X,Y)\in R^{2}\setminus S^{\text{in}}\;\text{and}\;\partial_{Y}\Phi =0\;\text{for }(X,Y)\in S^{\text{in}},$$

where $S^{\text{in}}=\{(X,Y):|X|\geq 1,Y=0\}$, i.e. the geometry looks locally planar as shown in figure 2, and we omit the subscript for $\Phi_{0}$ for clarity. Next we introduce the mapping W=arcsin⁡(Z) where Z=X+iY=Rexp⁡(iΘ) and W=U+iV, which transfers the problem of solving Laplace’s equation in $R^{2}\setminus S^{\text{in}}$ to solving Laplace’s equation in an infinitely extending strip, as shown in figure 2*b*, and as described by

3.3 $$(\partial_{U}^{2}+\partial_{V}^{2})\Phi_{D}=0\;\text{for }(U,V)\in D\;\text{and}\;\partial_{U}\Phi_{D}=0\;\text{for }U=\pm \frac{\pi }{2},$$

where D={(U,V):|U|≤π/2,V∈(−∞,∞)}. The appropriate solution is given by $\Phi_{D}=C_{1}\text{Re}(\text{i}W)+C_{2},$ where $C_{j}$ are as yet unknown, and we will see in the following sections why this form is the correct one for matching. Subsequently, the solution in the original domain follows as

3.4 $$\Phi =C_{1}\text{Re}(\text{i}\arcsin(Z))+C_{2}=C_{1}\text{Re}\left\{\log(\text{i}Z+\sqrt{1-Z^{2}})\right\}+C_{2},$$

where we define $\sqrt{1-Z^{2}}=\text{i}\sqrt{Z^{2}-1}$ .

### (a) Limiting behaviour of inner solution as $R=|X^{2}+Y^{2}{|}^{1/2}\to \infty$

We now require the field Φ as R→∞ in both the lower- and upper-half planes. To ensure single-valuedness, we introduce the double-angle representation

3.5a $$\sqrt{Z^{2}-1}=\sqrt{|Z^{2}-1|}{\text{e}}^{\text{i}(\Theta_{1}+\Theta_{2})/2},$$

over the cut plane $Z\in C\setminus B_{C}$ where $Z-1=R_{1}\exp(\text{i}\Theta_{1})$ and $Z+1=R_{2}\exp(\text{i}\Theta_{2})$ for $\Theta_{1}\in (-2\pi ,0)$ and $\Theta_{2}\in (-\pi ,\pi )$, with $B_{C}=\{(X,Y):X\in (\infty ,-1)\cup (1,\infty )\times Y=0\}$ denoting the branch cuts. Thus, if we proceed vertically to infinity in the upper-half plane (i.e. $\Theta_{1}\to -3\pi /2$ and $\Theta_{2}\to \pi /2$) and in the lower-half plane (i.e. $\Theta_{1}\to -\pi /2$ and $\Theta_{2}\to -\pi /2$) we obtain

3.5b $$\lim_{Z\to \text{i}\infty }\sqrt{Z^{2}-1}\approx -Z+\frac{1}{2Z}+O(Z^{-3})\;\text{and}\;\lim_{Z\to -\text{i}\infty }\sqrt{Z^{2}-1}\approx Z+O(Z^{-1}).$$

Accordingly the inner solution has, from (3.4), the asymptotic form

3.6 $$\lim_{R\to \infty }\Phi {|}_{R=\tilde{r}/\varepsilon }∼\left\{ \begin{array}{ll} -C_{1}\left[\log(\tilde{r})-\log\left(\frac{\varepsilon }{2}\right)\right]+C_{2}, & Z\in C^{U},\\ C_{1}\left[\log(\tilde{r})-\log\left(\frac{\varepsilon }{2}\right)\right]+C_{2}, & Z\in C^{L}, \end{array}\right.$$

where $C^{U}$ and $C^{L}$ denote the upper- and lower-half segments of the complex plane, respectively, and where we re-express the solution with respect to the original outer coordinate frame. We now proceed to the outer problem for our resonator array.

## 4. Outer problem formulation

The leading-order system for the outer problem is obtained by taking the limit ε→0 directly in the formulation (2.3), which returns a system with an identical structure to (2.3) but with an updated unit cell $\Omega \mapsto \Omega_{\text{out}}$, which is identical to Ω except for the resonator geometry replacement $S\mapsto S_{\text{out}}$, and the updated Neumann boundary conditions

4.1 $$\partial_{r}\phi =S_{0}\;\text{for}\;(x,y)\in S_{\text{out}},$$

where $S_{\text{out}}=(b\cos\theta ,b\sin\theta )$, with $\theta \in (0,2\pi )\setminus \theta_{0}$, with the aperture acting as a point source $S_{0}$ at $\theta =\theta_{0}$ with a yet to be determined amplitude. We now decompose the unit cell into two domains and consider a solution outside the resonator (the outer exterior solution $\phi_{\text{ext}}$) and inside the resonator (the outer interior solution $\phi_{\text{int}}$).

### (a) Outer exterior ansatz

In the region exterior to the resonator, but inside the fundamental unit cell, we pose the ansatz

4.2 $$\phi_{\text{ext}}=AH_{0}^{\left(1\right)}(\tilde{r})+\sum_{n=-\infty }^{\infty }\{a_{n}J_{n}(r)+b_{n}Y_{n}(r)\}{\text{e}}^{\text{i}n\theta },$$

where A, $a_{n}$ and $b_{n}$ are as yet unknown constants, ${\tilde{r}}^{2}=r^{2}+b^{2}-2rb\cos(\theta -\theta_{0})$, $J_{n}(z)$ and $Y_{n}(z)$ denote Bessel functions of the first and second kind, respectively, and $H_{n}^{\left(1\right)}(z)$ represents Hankel functions of the first kind. The impact of periodicity will be incorporated later in §4d. Next, we express the Neumann boundary condition (4.1) as

4.3 $$\partial_{r}\phi_{\text{ext}}{|}_{r=b}=S_{0}=\frac{C}{b}\delta (\theta -\theta_{0})=\frac{C}{2\pi b}\sum_{n=-\infty }^{\infty }{\text{e}}^{\text{i}n(\theta -\theta_{0})},$$

where C is unknown. The relationship between C and A is determined by applying Graf’s addition theorem [22, eqn (8.530)]

4.4 $$H_{0}^{\left(1\right)}(\tilde{r})=\left\{ \begin{array}{l} \sum_{n=-\infty }^{\infty }J_{n}(b)H_{n}^{\left(1\right)}(r){\text{e}}^{\text{i}n(\theta -\theta_{0})},\;r>b,\\ \sum_{n=-\infty }^{\infty }J_{n}(r)H_{n}^{\left(1\right)}(b){\text{e}}^{\text{i}n(\theta -\theta_{0})},\;r<b, \end{array}\right.$$

and taking the limit n→∞. By matching the Dirac delta singularity in (4.3) with the logarithmic singularity in the solution ansatz at $\left(b,\theta_{0}\right)$ in (4.2), we obtain the necessary form of the boundary condition C=2iA. Subsequently, after imposing the updated Neumann condition we obtain

4.5 $$\phi_{\text{ext}}=AH_{0}^{\left(1\right)}(\tilde{r})-\sum_{n=-\infty }^{\infty }\left\{\frac{AQ_{n}}{2J_{n}^{\prime }(b)}{\text{e}}^{-\text{i}n\theta_{0}}+\frac{Y_{n}^{\prime }(b)}{J_{n}^{\prime }(b)}b_{n}\right\}J_{n}(r){\text{e}}^{\text{i}n\theta }+\sum_{n=-\infty }^{\infty }b_{n}Y_{n}(r){\text{e}}^{\text{i}n\theta },$$

where $Q_{m}=J_{m}(b)H_{m}^{(1)\prime }(b)+J_{m}^{\prime }(b)H_{m}^{\left(1\right)}(b)$, and a prime denotes a derivative with respect to argument, i.e. $J_{m}^{\prime }(b)=\partial_{z}J_{m}(z){|}_{z=b}$. Note that the constants $b_{n}$ and A are as yet unknown, but that the form of $\phi_{\text{ext}}$ is prescribed.

### (b) Outer interior ansatz

Next we construct a corresponding form of the outer solution inside the resonator following an identical procedure to the above. Hence, we write

4.6 $$\phi_{\text{int}}=BH_{0}^{\left(1\right)}(\tilde{r})+\sum_{n=-\infty }^{\infty }f_{n}J_{n}(r){\text{e}}^{\text{i}n\theta },$$

where *B* and *f*_*n*_ are as yet unknown constants, and consider the Neumann boundary condition $\partial_{r}\phi_{\text{int}}{|}_{r=b}=D\delta (\theta -\theta_{0})/b$, where D is once more unknown. Imposing the Neumann condition above, using Graf’s addition theorem (4.4), and considering the limit n→∞, we find that D=−2iB on matching divergent terms. Imposing the updated Neumann condition yields

4.7 $$\phi_{\text{int}}=BH_{0}^{\left(1\right)}(\tilde{r})-\frac{B}{2}\sum_{n=-\infty }^{\infty }\frac{Q_{n}}{J_{n}^{\prime }(b)}J_{n}(r){\text{e}}^{\text{i}n(\theta -\theta_{0})},$$

where B remains unknown. We can now take the outer solutions in the exterior (4.5) and interior (4.7) domains, and determine their asymptotic forms as we approach the aperture

4.8 limθ→θ0limr→bϕ∼{2iAπ[γe−iπ2+log⁡(r~2)]+∑n=−∞∞bnYn(b)einθ0−∑n=−∞∞{AQn2+bnYn′(b)einθ0}Jn(b)Jn′(b),r↓b,2iBπ[γe−iπ2+log⁡(r~2)]−B2∑n=−∞∞QnJn′(b)Jn(b),|br↑b,

where $\gamma_{\text{e}}$ denotes the Euler–Mascheroni constant. Having determined partial solutions (up to an infinite set of constants) for both the inner and outer solutions, and their asymptotic representations near the aperture, we now proceed to asymptotic matching.

### (c) Matched asymptotics procedure with partial solutions

The unknown terms A, B, $C_{1}$ and $C_{2}$ in the above are obtained by matching terms (to leading order) from the inner and outer solution representations in the following limit [7,8]

4.9 $$\lim_{\theta \to \theta_{0}}\lim_{r\to b}\phi =\lim_{R\to \infty }\Phi {|}_{R=r/\varepsilon },$$

where the asymptotic forms are given above in (4.8) and (3.6). Specifically, we match the inner and outer solutions, in the interior/lower and exterior/upper regions, and then compare logarithmic and non-logarithmic terms to obtain a system of equations. These yield B=−A and

4.10 $$A=\frac{2}{\pi bh_{\varepsilon }}\sum_{n=-\infty }^{\infty }\frac{b_{n}}{J_{n}^{\prime }(b)}{\text{e}}^{\text{i}n\theta_{0}},\;\text{where }h_{\varepsilon }=\frac{4\text{i}}{\pi }\left[\gamma_{\text{e}}-\frac{\text{i}\pi }{2}+\log\left(\frac{\varepsilon }{4}\right)\right]-\sum_{n=-\infty }^{\infty }\frac{Q_{n}J_{n}(b)}{J_{n}^{\prime }(b)}.$$

### (d) Lattice contributions and asymptotic resonator system

The final step in our derivation of an eigenvalue problem for the resonant array involves returning to the exterior solution ansatz (4.5) and applying Graf’s addition theorem (4.4) to obtain

4.11 $$\begin{array}{rl} \phi_{\text{ext}} & \;=\sum_{n=-\infty }^{\infty }\left[AJ_{n}(b){\text{e}}^{-\text{i}n\theta_{0}}-\frac{A}{2}\frac{Q_{n}}{J_{n}^{\prime }(b)}{\text{e}}^{-\text{i}n\theta_{0}}-\frac{Y_{n}^{\prime }(b)}{J_{n}^{\prime }(b)}b_{n}\right]J_{n}(r){\text{e}}^{\text{i}n\theta }\\ & \;\;+\sum_{n=-\infty }^{\infty }[\text{i}AJ_{n}(b){\text{e}}^{-\text{i}n\theta_{0}}+b_{n}]Y_{n}(r){\text{e}}^{\text{i}n\theta }=\sum_{n=-\infty }^{\infty }\{c_{n}J_{n}(r)+d_{n}Y_{n}(r)\}{\text{e}}^{\text{i}n\theta }, \end{array}$$

where the $c_{n}$ and $d_{n}$ coefficients are related through the dynamic Rayleigh identity [4, eqn (3.119)]

4.12 $$c_{n}=\sum_{m=-\infty }^{\infty }{\left(-1\right)}^{m+n}S_{m-n}^{Y}({\text{k}}_{B})d_{m},$$

which follows from an application of Green’s second identity inside the unit cell. The Rayleigh identity incorporates multiple scattering contributions from neighbouring cells by imposing the Bloch conditions (4.1). Expressions for the lattice sums $S_{m}^{Y}$ are given in appendix A for reference.

At this point, we possess an identity relating $c_{n}$ and $d_{n}$ in (4.12), expressions for $c_{n}$ and $d_{n}$ in terms of A and $b_{n}$ in (4.11), and a relation between A and $b_{n}$ from the matched asymptotics procedure (4.10). Combining all of these expressions, we obtain the eigenvalue problem

4.13 $$\begin{array}{rl} & g_{n}+\sum_{m=-\infty }^{\infty }{\left(-1\right)}^{m+n}S_{m-n}^{Y}({\text{k}}_{B})\frac{J_{m}^{\prime }(b)}{Y_{n}^{\prime }(b)}{\text{e}}^{-\text{i}(m-n)\theta_{0}}g_{m}\\ & \;\;+\frac{\text{i}}{\pi bh_{\varepsilon }}\left(\sum_{q=-\infty }^{\infty }g_{q}\right)\left(E_{n}+2\sum_{m=-\infty }^{\infty }{\left(-1\right)}^{n+m}S_{m-n}^{Y}({\text{k}}_{B})\frac{J_{m}(b)}{Y_{n}^{\prime }(b)}{\text{e}}^{-\text{i}(m-n)\theta_{0}}\right)=0, \end{array}$$

which must be satisfied for all n∈Z, where

4.14 $$b_{n}=J_{n}^{\prime }(b)g_{n}{\text{e}}^{-\text{i}n\theta_{0}}\;\text{and}\;E_{n}=\frac{J_{n}^{\prime }(b)Y_{n}(b)+Y_{n}^{\prime }(b)J_{n}(b)}{J_{n}^{\prime }(b)Y_{n}^{\prime }(b)}.$$

In the limit as the gap is closed ε→0 then $h_{\varepsilon }\to -\text{i}\infty$ and we recover the conventional system for an array of homogeneous Neumann cylindrical inclusions [4, eqn (3.120)]. Next, for numerical and analytical purposes, we require that the infinite dimensional system (4.13), and all sums contained therein, are truncated in order to obtain a finite-dimensional system; imposing a vanishing determinant condition then yields the dispersion equation for that truncation (denoted by the truncation level L). In general, the accuracy of the determinant is improved as we increase the truncation level to include higher orders. For reference, care must be taken for large L as accurate band diagrams may be constructed but inaccurate modal fields may arise (i.e. from (4.5) and (4.7)) as errors in the asymptotic approximations could become significant.

It is well known that for periodic problems involving cylinders with Neumann boundary conditions, a lowest order (monopole) truncation is unable to accurately describe the eigenstate at low frequencies. As such, we proceed to the next section by considering a dipolar truncated system.

### (e) Leading-order approximation to the dispersion equation

Considering the system (4.13) we now truncate all sums, and consider all orders, to within a dipole approximation L=1 (i.e. keeping terms n=−1,0,1) to construct the dipole system. We then evaluate Taylor series in the small b (long wavelength) limit to obtain the leading-order system ${\text{A}}_{0}\text{g}=0$ given by

4.15 $$\begin{array}{rl} & \left[\begin{array}{ccc} 1+\frac{1}{4}\pi b^{2}S_{0}^{Y} & -\frac{1}{h_{\varepsilon }}\text{i}bS_{1}^{Y*}{\text{e}}^{\text{i}\theta_{0}}+\frac{1}{4}\pi b^{3}S_{1}^{Y*}{\text{e}}^{\text{i}\theta_{0}} & -\frac{1}{4}\pi b^{2}S_{2}^{Y*}{\text{e}}^{2\text{i}\theta_{0}}\\ -\frac{1}{4}\pi bS_{1}^{Y}{\text{e}}^{-\text{i}\theta_{0}} & -\frac{2\text{i}}{\pi b^{2}h_{\varepsilon }}+\frac{\text{i}S_{0}^{Y}}{h_{\varepsilon }}+1-\frac{1}{4}\pi b^{2}S_{0}^{Y} & \frac{1}{4}\pi bS_{1}^{Y*}{\text{e}}^{\text{i}\theta_{0}}\\ -\frac{1}{4}\pi b^{2}S_{2}^{Y}{\text{e}}^{-2\text{i}\theta_{0}} & \frac{1}{h_{\varepsilon }}\text{i}bS_{1}^{Y}{\text{e}}^{-\text{i}\theta_{0}}-\frac{1}{4}\pi b^{3}S_{1}^{Y}{\text{e}}^{-\text{i}\theta_{0}} & 1+\frac{1}{4}\pi b^{2}S_{0}^{Y} \end{array}\right]\left[\begin{array}{c} g_{1}\\ g_{0}\\ g_{-1} \end{array}\right]=\left[\begin{array}{c} 0\\ 0\\ 0 \end{array}\right], \end{array}$$

and where we have made use of the lattice sum asymptotic forms [4, eqns (3.132)–(3.134)] in the long wavelength and low frequency limits

4.16 $$\lim_{k_{B}\to \Gamma }\lim_{k\to 0}\{S_{0}^{Y},S_{1}^{Y},S_{2}^{Y}\}∼\left\{-\frac{4}{d^{2}}\frac{1}{k_{B}^{2}-1},-\frac{4\text{i}k_{B}}{d^{2}}\frac{{\text{e}}^{\text{i}\theta_{B}}}{k_{B}^{2}-1},\frac{4k_{B}^{2}}{d^{2}}\frac{{\text{e}}^{2\text{i}\theta_{B}}}{k_{B}^{2}-1}\right\},$$

with $\left(k_{B},\theta_{B}\right)$ representing the polar form of the Bloch vector ${\text{k}}_{B}$ and ∗ denoting the complex conjugate operation. In the system above, we have also made use of the dominant balance scaling d=O(b), to avoid implicitly examining the vanishing filling fraction $f=\pi b^{2}/d^{2}$ limit as b→0, and also used the dominant balance scaling $\log(\varepsilon /(2b))=O(b^{-2})$ appearing in

4.17a $$\lim_{b\to 0}h_{\varepsilon }∼\frac{4\text{i}}{\pi b^{2}}\left[1-\frac{b^{2}}{8}+b^{2}\log\left(\frac{\varepsilon }{2b}\right)\right]=\frac{4\text{i}}{\pi b^{2}}f_{\varepsilon }.$$

Next we introduce the substitutions

4.17b $$S_{0}^{Y}=\frac{4}{\pi b^{2}}A_{0},\;S_{1}^{Y}=\frac{4\text{i}}{\pi b^{2}}A_{1}{\text{e}}^{\text{i}\theta_{B}},\;\mathrm{and}\;S_{2}^{Y}=\frac{4}{\pi b^{2}}A_{2}{\text{e}}^{2\text{i}\theta_{B}},$$

where $A_{0}$, $A_{1}$ and $A_{2}$ are strictly real, and evaluate the determinant of the system (4.15) to obtain the leading-order dispersion equation

4.18 $$\begin{array}{rl} & \;(1+A_{0}+A_{2})\left\{\left(1-\frac{1}{f_{\varepsilon }}\right)[A_{0}(1+A_{0}-A_{2})-2A_{1}^{2}]-\left(1-\frac{1}{2f_{\varepsilon }}\right)(1+A_{0}-A_{2})\right\}=0, \end{array}$$

and so after returning to the forms for $S_{m}^{Y}$ in (4.16) once more we obtain the lowest-order approximation for the dispersion equation of the first band in the form

4.19 $$k_{B}^{2}=\frac{1+f}{1-f}\left(1-\frac{2f(1-f_{\varepsilon })}{1-2f_{\varepsilon }}\right),\;\text{where we repeat that }f=\frac{\pi b^{2}}{d^{2}}.$$

In the limit of vanishing aperture, we have that $f_{\varepsilon }\to \infty$ and subsequently we recover the classical result for an array of Neumann cylinders in the form $k_{B}^{2}=1+f$ [4, eqn (3.158)]. Thus, by specifying purely geometric parameters, namely the radius $\bar{b}$, half-angle $\theta_{\mathrm{ap}}$ and filling fraction f, as well as the Bloch wave vector ${\bar{\text{k}}}_{B}$, it is then possible to solve for k in (4.19) and obtain the leading-order approximation to the first band surface over the entire Brillouin zone. Note that the absence of $\theta_{\text{B}}$ in the above means that the leading-order approximation is unable to describe the low-frequency anisotropy present in the first band. For this reason, we proceed to a first-order correction, but first discuss some of the features of the approximation (4.19). For example, by substituting the leading-order behaviour

4.20 $$f_{\varepsilon }=\left[1-\frac{b^{2}}{8}+b^{2}\log\left(\frac{\varepsilon }{2b}\right)\right]∼1+b^{2}\log\left(\frac{\theta_{\text{ap}}}{2}\right),$$

into the dispersion equation (4.19), we obtain the result presented in Llewellyn Smith & Davis [10], which we correct for an errant sign error to:

4.21 $$2b^{2}\log\left(\frac{2}{\theta_{\text{ap}}}\right)=\frac{k_{B}^{2}-(1+f)/(1-f)}{k_{B}^{2}-(1+f)}.$$

Following the discussion in Llewellyn Smith & Davis [10], under the limit of vanishing aperture, $\theta_{\mathrm{ap}}\to 0$, the representation (4.21) returns the classical result for an array of Neumann cylinders as given above. Likewise in the low-frequency limit ω→0 we obtain a lowest-order dispersion relation for our Helmholtz resonator array [10] in the form

4.22 $$k_{B}^{2}=\frac{1+f}{1-f},\;\text{ or }\;\omega =\sqrt{\frac{B}{\rho }}\sqrt{\frac{1-f}{1+f}}\;{\bar{k}}_{B},$$

however numerical investigations show this leading-order result to be accurate along one high-symmetry direction alone. As a final remark on the leading-order dispersion equation (4.19), we note that although it is unable to correctly describe the first band, it is able to approximate the cut-off frequency of the first band to within moderate accuracy (i.e. the maximum eigenfrequency of the first band but not necessarily the lower bound on the first band gap). This is done using the vanishing denominator condition in (4.19) to obtain

4.23 $$k_{\text{max}}\approx \frac{2}{\bar{b}}{\left[1+8\log\left(\frac{2}{\theta_{\text{ap}}}\right)\right]}^{-1/2},\;\text{ or }\;\omega_{\text{max}}\approx \frac{2c_{p}}{\bar{b}}{\left[1+8\log\left(\frac{2}{\theta_{\text{ap}}}\right)\right]}^{-1/2}.$$

The above expression may also be used to determine the configuration of the resonator within the unit cell, for example, if we seek a resonance in air $c_{p}=\sqrt{B/\rho }=343.21\;m\cdot s^{-1}$ at the frequency $f_{\text{max}}=60\;\mathrm{Hz}$ with the (arbitrary) design restriction of the half aperture length being $\bar{\ell }=0.01$ m=1 cm, then we require a radius of $\bar{b}=0.312$ m=31.2 cm.

### (f) First-order correction to the dispersion equation

The first-order correction to the system (4.15) within a dipolar truncation takes the form

4.24a Bg=0,

where $\text{B}={\text{A}}_{0}+{\text{A}}_{1}$, with ${\text{A}}_{0}$ given in (4.15), and

4.24b $${\text{A}}_{1}=\left[\begin{array}{ccc} -\frac{\text{i}b}{h_{\varepsilon }}S_{1}^{Y*}{\text{e}}^{\text{i}\theta_{0}} & \frac{\text{i}b^{2}}{2h_{\varepsilon }}(S_{0}^{Y}-S_{2}^{Y*}{\text{e}}^{2\text{i}\theta_{0}}) & -\frac{\text{i}b}{h_{\varepsilon }}S_{1}^{Y*}{\text{e}}^{\text{i}\theta_{0}}\\ -\frac{2\text{i}}{\pi b^{2}h_{\varepsilon }}+\frac{\text{i}}{h_{\varepsilon }}S_{0}^{Y} & \frac{\text{i}b}{2h_{\varepsilon }}(S_{1}^{Y*}{\text{e}}^{\text{i}\theta_{0}}-S_{1}^{Y}{\text{e}}^{-\text{i}\theta_{0}}) & -\frac{2\text{i}}{\pi b^{2}h_{\varepsilon }}+\frac{\text{i}}{h_{\varepsilon }}S_{0}^{Y}\\ \frac{\text{i}b}{h_{\varepsilon }}S_{1}^{Y}{\text{e}}^{-\text{i}\theta_{0}} & \frac{\text{i}b^{2}}{2h_{\varepsilon }}(S_{0}^{Y}-S_{2}^{Y}{\text{e}}^{-2\text{i}\theta_{0}}) & \frac{\text{i}b}{h_{\varepsilon }}S_{1}^{Y}{\text{e}}^{-\text{i}\theta_{0}} \end{array}\right].$$

Solving for detB=0 we obtain the principal result of this paper:

4.25a $$k_{B}^{2}=\frac{\left(f+1)[b^{2}f(2f-1)+f_{\varepsilon }(f+1)(2f_{\varepsilon }f-2f_{\varepsilon }-2f+1)\right]}{b^{2}f\cos(2[\theta_{0}-\theta_{B}])+b^{2}f^{2}+f_{\varepsilon }(2f_{\varepsilon }-1)(f^{2}-1)},$$

which is an asymptotic dispersion equation implicitly describing the first spectral band surface. The expression (4.25*a*) is crucially able to capture the low-frequency anisotropy (i.e. differing low-frequency slopes) present in the first spectral band. In the closed aperture limit $f_{\varepsilon }\to \infty$, we recover the result for an array of perfect Neumann cylinders $k_{B}^{2}=1+f$ from (4.25*a*). We now briefly discuss the features of the dispersion relation derived above; note the presence of all angular dependencies: $\theta_{0}$, $\theta_{\mathrm{ap}}$ (via $f_{\varepsilon }$), and $\theta_{B}$, and that the analogue to (4.21) is considerably less compact as $f_{\varepsilon }^{2}$ terms are present. Rearranging (4.25*a*) we obtain a low-frequency dispersion relation of the form $\omega =c_{p}^{\text{eff}}(\theta_{B},\omega ){\bar{k}}_{B}$ where

4.25b $$c_{p}^{\text{eff}}(\theta_{B},\omega )=\sqrt{\frac{B}{\rho }}\sqrt{\frac{b^{2}f\cos(2[\theta_{0}-\theta_{B}])+b^{2}f^{2}+f_{\varepsilon }(2f_{\varepsilon }-1)(f^{2}-1)}{\left(f+1)[b^{2}f(2f-1)+f_{\varepsilon }(f+1)(2f_{\varepsilon }f-2f_{\varepsilon }-2f+1)\right]}},$$

in which the effective wave speed $c_{p}^{\text{eff}}$ possesses dependence on both the frequency and Bloch vector direction (i.e. exhibits both temporal and spatial dispersion). By differentiating (4.25*a*) the group velocity is obtained explicitly but is not included here for compactness. A detailed discussion of homogenization methods is made in Part II of this work [23].

## 5. Numerical results

In this section, we compare results from a full finite-element treatment for the original array problem (2.3) against results from the multipole-matched asymptotic system (4.13), as well as the leading order (4.19) and first-order (4.25*a*) dispersion equation approximations. The objective is to examine the impact of varying the central aperture angle $\theta_{0}$, the filling fraction f and the half-angle aperture width $\theta_{\mathrm{ap}}$ upon the first band and first band gap. We use finite-element results obtained from existing and readily available software to independently benchmark the multipole-matched asymptotic results obtained here.

**Figure 3.**
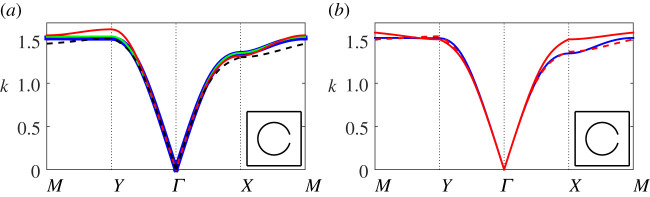
Band diagrams for a square array of thin-walled Helmholtz resonators comparing: (*a*) the finite-element solution (blue line) with results from the system (4.13) within a dipole truncation using either the convergent forms for $S_{m}^{Y}$ (A 2) (red line) or the asymptotic forms for $S_{m}^{Y}$ (4.16) (dashed black line), and results from (4.13) within a quadrupolar truncation (green line); (b) the full finite-element solution (blue line) with the symmetric lowest-order approximation (4.19) (solid red line) and the first-order correction (4.25*a*) (dashed red line). In both figures we specify $\bar{b}=0.3$, $\bar{d}=1$, $\theta_{0}=0$, $\theta_{\mathrm{ap}}=\pi /12$ and inset the unit cell. (Online version in colour.)

In figure 3, we examine the first band surface of a representative resonator array possessing a moderate half angle $\theta_{\mathrm{ap}}=\pi /12$, apertures located at $\theta_{0}=0$ and filling fraction $f=\pi b^{2}/d^{2}\approx 0.28$: in figure 3*a* we compare results for the first band over the edge of the irreducible Brillouin zone (highlighted in figure 1*b*) using both finite-element methods and our multipole-matched asymptotic system (4.13). Key features of the first band include different low-frequency slopes along the high symmetry directions ΓX and ΓY, the existence of an almost flat band at the cut-off frequency along MY and a saddle point frequency located at X. In this representative example, we find that the system (4.13) is able to describe the first band well over its entire frequency range (solid red line) within a dipole truncation, even with the use of lattice sum approximations (dashed black line), demonstrating that although the full system overestimates the frequency at Y, a dipole truncation still gives a reasonable approximation over the entire Brillouin zone. Also superposed is the result within a quadrupolar truncation (solid green) which is an excellent approximation over the entire range, emphasizing the importance of quadrupolar contributions to the modes as we approach the band edge. The adjacent figure 3*b* overlays the finite-element result (blue line) with the lowest (solid red line) and first-order (dashed red line) approximations for the dispersion equation. As described earlier, the lowest-order approximation is symmetric along all high symmetry directions (i.e. returns an isotropic approximation to the array), but is accurate only along ΓY, being unable to capture the saddle point at X; an improved description is obtained using the first-order approximation, with only a minor discrepancy in the band curvature along the XM direction. In summary, the discrepancies in curvature along XM are due to an absence of quadrupolar terms, the series expansions for the Bessel functions, and the lattice sum approximations, as shown in figure 3*a*.

**Figure 4.**
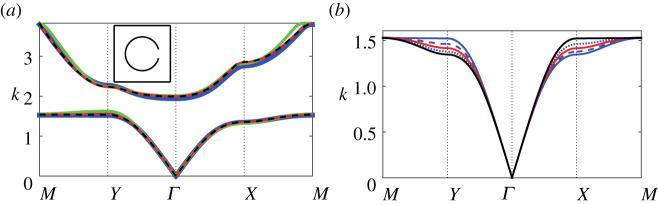
Band diagrams for a square array of thin-walled Helmholtz resonators as: (*a*) we increase the truncation of the multipole system (4.13) from dipolar (green line), to quadrupolar (red line) and sextapolar (dashed black line), with finite-element result superposed (blue line) and fundamental unit cell inset; (*b*) the central aperture angle is varied: $\theta_{0}=0$ (blue line), $\theta_{0}=\pi /6$ (dashed blue line), $\theta_{0}=\pi /4$ (red line), $\theta_{0}=\pi /3$ (dotted blue line) and $\theta_{0}=\pi /2$ (black line) with curves calculated using finite-element methods. In both figures we use $\bar{b}=0.3$, $\bar{d}=1$ and $\theta_{\mathrm{ap}}=\pi /12$. (Online version in colour.)

In figure 4*a*, we compute the first two bands for the same resonator array configuration used in figure 3 using both finite-element and our multipole-matched asymptotic expansion method (4.13); we observe reasonable qualitative descriptions at dipolar truncation over both bands, with improvements in convergence over the first band along the YM and XM symmetry paths for quadrupolar truncations and higher. We observe that very good convergence for the (approximate) multipole-matched asymptotic treatment is achieved at quadrupolar truncation, and although the multipole-matched asymptotic system does not converge precisely to the finite-element result, it still performs extremely well for an approximate description. Importantly, this figure suggests that the width of the first band gap may be determined with reasonable accuracy (with high enough truncation L), and that the greatest discrepancies in our model are observed on second band at the highest frequencies, as might be expected. In figure 4*b*, we consider the effect of varying the central aperture angle $\theta_{0}$ on the band structure curvature (over the irreducible Brillouin zone for a high frequency configuration); results for several configurations in the range $0\leq \theta_{0}\leq \pi /2$ are superposed where we observe only small changes in the curvature of the first band for different $\theta_{0}$ angles. Results from our multipole formulation match those obtained using finite element methods, as expected, but are excluded here to avoid figure overcrowding. Accordingly, we consider $\theta_{0}=0$ in all other numerical results. For $\theta_{0}=\pi /4$ we recover a symmetric band surface where the lowest order approximation (4.19) possesses identical symmetry, however it overestimates frequencies at X and M; see figure 3*b*.

**Figure 5.**
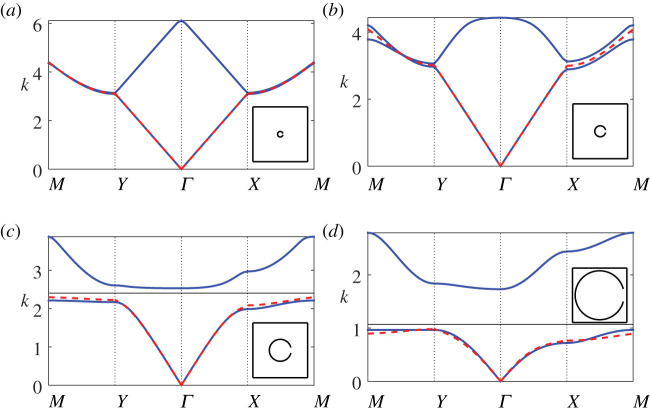
Band diagrams for a square array of thin-walled Helmholtz resonators as the radius is varied: (*a*) $\bar{b}=0.05$, (*b*) $\bar{b}=0.1$, (*c*) $\bar{b}=0.2$ and (*d*) $\bar{b}=0.45$ with fundamental unit cells inset. Blue lines denote from finite-element solution, red dashed lines denote results from the first-order correction (4.25*a*) and black lines denote estimates for the edge of the band gap (4.23). In the above figures, we use $\bar{d}=1$, $\theta_{0}=0$ and $\theta_{\mathrm{ap}}=\pi /12$. (Online version in colour.)

In figure 5, we examine the performance of our first-order description (4.25*a*) as the filling fraction is varied, for the same configuration as in figure 3 but as we vary the radius $\bar{b}$. We also superpose the estimate for the cut-off (band edge) frequency (4.23) for instances where a band gap exists. We observe that the description for the first band works well both in the presence (here, $\bar{b}>0.1$) and absence (here, $\bar{b}<0.1$) of a band gap, although at higher filling fractions, the first-order description is unable to capture the cut-off frequency and the curvature with extreme precision, as we approach the M point. In this figure we include the first two bands to examine also the effect of filling fraction on the first band gap; we find that the gap width increases as f increases, for fixed aperture width. Interestingly, the estimate for the cut-off frequency works best at moderate-to-high filling fractions (i.e. f>0.28), and that at very dilute filling fractions the bands approach the dispersion curves for free-space, despite the presence of a resonator. Note that it is possible to determine an upper bound on the width of the first band gap by specifying the Bloch coordinate to lie at the Γ, X, Y and M points, solving for vanishing determinant, choosing the second eigenvalue at these coordinates, and then selecting the minimum of these values. We advise solving the full system (4.13) to obtain these values and advise against the use of the dispersion equation (4.25*a*) for this purpose, as the accuracy of the second band values are not always assured within the description.

**Figure 6.**
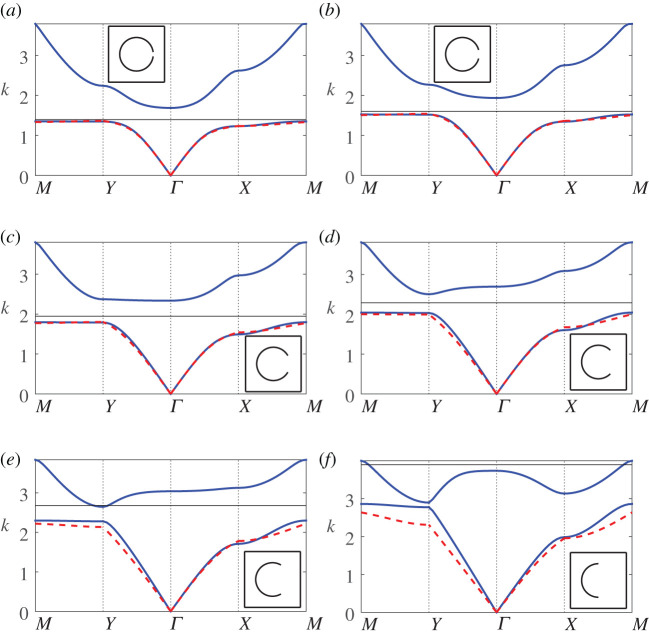
Band diagrams for a square array of thin-walled Helmholtz resonators as the aperture half-angle is varied: (*a*) $\theta_{\mathrm{ap}}=\pi /24$, (*b*) $\theta_{\mathrm{ap}}=\pi /12$, (*c*) $\theta_{\mathrm{ap}}=\pi /6$, (*d*) $\theta_{\mathrm{ap}}=\pi /4$, (*e*) $\theta_{\mathrm{ap}}=\pi /3$, (*f* ) $\theta_{\mathrm{ap}}=\pi /2$ with fundamental unit cells inset. Figure legends are identical to those in figure 5. In the above figures, we use $\bar{d}=1$, $\theta_{0}=0$ and $\bar{b}=0.3$. (Online version in colour.)

In figure 6, we investigate how well the first-order description (4.25*a*) works with increasing aperture size, that is, we examine the same configuration as in figure 3 but now vary the half-angle $\theta_{\mathrm{ap}}$. We find that our description works well up to half-angles of $\theta_{\mathrm{ap}}\approx \pi /4$, which is perhaps remarkable for a system formally derived in the vanishing aperture limit. We observe that the first-order description breaks down when a clear minimum of the second band surface appears at the Y high-symmetry coordinate, rather than at the Γ point. It also corresponds with the estimated band maximum appearing at approximately the midpoint of the band gap, which closes with increasing aperture size. Finally, we observe that our description still holds moderately well up to a larger half-angle of $\theta_{\mathrm{ap}}=\pi /3$, along the ΓX direction.

Having examined the parameter ranges over which our system and dispersion equation are valid, we now investigate the effects of wall thickness on results for Helmholtz resonator arrays.

## 6. Extension to thick-walled resonators

**Figure 7.**
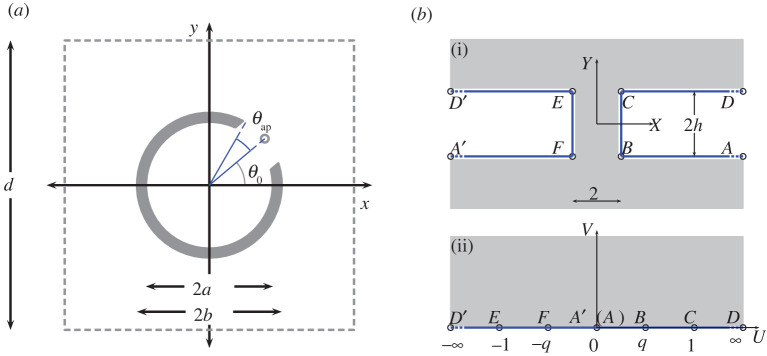
(*a*) Fundamental unit cell for a square array of period d containing a thick-walled cylindrical resonator with inner radius a, outer radius b and an aperture centred at $\theta_{0}$ with half-angle $\theta_{\mathrm{ap}}=\ell /b$, where the outer aperture arc length is 2ℓ; (*b*)(i) inner problem geometry with unbounded polygonal (fluid) domain overlaid in grey; (*b*)(ii) inner problem geometry obtained via the Schwarz–Christoffel mapping (6.3*a*); the capital letters A,…,D and $A^{\prime },\ldots ,D^{\prime }$ denote the points of correspondence in the Z(=X+iY) and W(=U+iV) complex planes. (Online version in colour.)

We now pose the governing equations for the thick-walled resonator problem shown in figure 7*a*, in terms of the non-dimensional coordinates given in (2.2). By thick-walled, we mean a cylinder whose aperture arc length, $2\bar{\ell }$, is of the same order as its thickness. This has an identical structure to (2.3) earlier but with $S\mapsto S_{T}$ and a modified Neumann boundary condition of the form

6.1a $$\partial_{r}\phi =0\;\text{for }(x,y)\in S_{T},$$

where $S_{T}$ denotes the thick-walled Helmholtz resonator. The definition of $S_{T}$ is chosen to ensure that the resonator walls in the neck are parallel to one another, as shown in figure 7*a*, and admits the inner problem domain presented in figure 7*b*. We write the non-dimensional inner radius a=b−2hε, where ε=ℓ is the aperture arc half-length at the outer radius b, and h is the aspect ratio of the channel (resonator neck). Note also that the definition of the inner radius given above implicitly treats the aperture neck length (b−a) as O(ε), which later ensures that the filling fraction is held constant (see below).

### (a) Inner problem formulation

As before, we first rotate and translate the array via the operation $\left(\tilde{x},\tilde{y})\mapsto (x\sin\theta_{0}-y\cos\theta_{0},x\cos\theta_{0}+y\sin\theta_{0}-b+\ell h\right)$ so that the origin in tilde coordinates is centred and oriented on the aperture in the fundamental cell. As in §3, we introduce the inner scaling (3.1) and a regular expansion for ϕ to obtain the leading-order system

6.2 $$(\partial_{X}^{2}+\partial_{Y}^{2})\Phi =0\;\text{for }(X,Y)\in R^{2}\setminus S_{T}^{\text{in}},\;\text{with}\;\partial_{N}\Phi =0\;\text{for }(X,Y)\in S_{T}^{\text{in}},$$

where $\partial_{N}$ denotes the normal derivative, the resonator walls are defined by $S_{T}^{\text{in}}=\{(X,Y):|X|\geq 1\times Y\in [-h,h]\}$ as shown in figure 7*b* and we omit the subscript for $\Phi_{0}$. Next we introduce the Schwarz–Christoffel mapping [24] between the Z and W planes:

6.3a $$Z(W)=\frac{\int_{1}^{W}\varkappa (\zeta )\;\text{d}\zeta +\int_{-q}^{W}\varkappa (\zeta )\;\text{d}\zeta }{\int_{-q}^{q}\;\varkappa (\zeta )\;\text{d}\zeta },\;\text{where}\;\varkappa (\zeta )=\frac{\sqrt{\left(\zeta^{2}-1)(\zeta^{2}-q^{2}\right)}}{\zeta^{2}},$$

in which the parameter q is related exactly to the specified aspect ratio h via

6.3b $$h=\frac{1}{2}{\left[2E(q^{2})+(q^{2}-1)K(q^{2})\right]}^{-1}[-2E(1-q^{2})+(1+q^{2})K(1-q^{2})],$$

and E(k) and K(k) are complete elliptic integrals of the first and second kind, respectively. Note that the aspect ratio h cannot be too high as q vanishes exponentially in the limit of large h (for example, for h=4 we have $q\approx 1.8879\times 10^{-6}$), which may cause accuracy issues from a numerical perspective. Hence, the treatment we outline here implicitly assumes thick-walled resonators where the channel aspect ratio h is not too high (in fact, we may consider it to be O(1)).

Subsequently, using (6.3*a*) we map the problem of solving Laplace’s equation in the physical junction domain $R_{2}\setminus S_{T}^{\text{in}}$ shown in figure 7*b* to solving Laplace’s equation in the upper-half plane of the W-plane shown in figure 7*b*(ii), where a vanishing Neumann condition is imposed along the real line. For the latter problem, we may immediately offer a solution in the form $\Phi (W)=C_{3}\text{Re}\{\log W\}+C_{4}$, where from the leading-order asymptotic form for the mapping (6.3*a*)

6.4 $$\lim_{W\to 0}Z(W)∼\frac{C(q)q}{W}\;\text{and}\;\lim_{W\to \infty }Z(W)∼C(q)W,$$

we obtain the leading-order result in the original inner region as

6.5 $$\lim_{R\to \infty }\Phi {|}_{\left\{R=\tilde{r}/\varepsilon ,R=\overset{ˇ}{r}/\varepsilon \right\}}∼\left\{ \begin{array}{ll} C_{3}\;\text{Re}[\log(\tilde{r})-\log\{C(q)\varepsilon \}]+C_{4}, & Z\in C^{U},\\ C_{3}\;\text{Re}[\log\{q\;C(q)\varepsilon \}-\log(\overset{ˇ}{r})]+C_{4}, & Z\in C^{L}, \end{array}\right.$$

where

6.6 $$C(q)={\left(2E(q^{2})+(q^{2}-1)K(q^{2})\right)}^{-1}\;\text{and}\;\overset{ˇ}{r}=\sqrt{{\left(x-a\cos\theta_{0}\right)}^{2}+{\left(y-a\sin\theta_{0}\right)}^{2}},$$

and we reintroduce tilde notation as before. Note that when q=1 we have C=1/2 and h=0 to recover the asymptotic form for the thin-walled resonator outlined before. Next we outline modifications to the outer problem, specifically, the outer interior problem solution.

### (b) Outer problem formulation

The outer exterior solution is unchanged, and the outer interior solution proceeds analogously to that given in §4b, but with the replacement coordinates and parameters $\tilde{r}\mapsto \overset{ˇ}{r}$, b↦a and $Q_{m}\mapsto {\overset{ˇ}{Q}}_{m}$, where ${\overset{ˇ}{Q}}_{m}=J_{m}(a)H_{m}^{(1)\prime }(a)+J_{m}^{\prime }(a)H_{m}^{\left(1\right)}(a)$. Hence, as we approach the resonator neck from the interior and exterior domains, the outer solution now takes the form

6.7 limθ→θ0limr→b,aϕout∼{2iAπ[γe−iπ2+log⁡(r~2)]+∑n=−∞∞bnYn(b)einθ0−∑n=−∞∞{AQn2+bnYn′(b)einθ0}Jn(b)Jn′(b),r↓b,2iBπ[γe−iπ2+log⁡(rˇ2)]−B2∑n=−∞∞QˇnJn′(a)Jn(a),|br↑a,

which is the analogue to the thin-walled expression in (4.8) given earlier, but with the addition of the inner wall radius a=b−2hε.

### (c) Matching procedure for thick-walled resonators

Having obtained the inner and outer asymptotic representations (6.5) and (6.7), we now match inner fields at infinity, in the upper- and lower-half planes, to outer fields as we approach either side of the neck r↓b and r↑a, respectively. As before, after matching logarithmic and non-logarithmic terms we obtain a system of equations, from which we find that B=−A once more, but obtain an updated relationship between A and $b_{n}$ analogous to that given in (4.10) but with the replacement $h_{\varepsilon }\mapsto {\overset{ˇ}{h}}_{\varepsilon }$ where

6.8 $${\overset{ˇ}{h}}_{\varepsilon }=\frac{4\text{i}}{\pi }\left[\gamma_{\text{e}}-\frac{\text{i}\pi }{2}+\log\left(\frac{\varepsilon C(q)\sqrt{q}}{2}\right)\right]-\frac{1}{2}\sum_{n=-\infty }^{\infty }\frac{Q_{n}J_{n}(b)}{J_{n}^{\prime }(b)}-\frac{1}{2}\sum_{n=-\infty }^{\infty }\frac{{\overset{ˇ}{Q}}_{n}J_{n}(a)}{J_{n}^{\prime }(a)}.$$

Thus, we obtain an eigenvalue problem for the thick-walled resonator case that is identical to (4.13), but with the simple replacement $h_{\varepsilon }\mapsto {\overset{ˇ}{h}}_{\varepsilon }$. This highlights a significant advantage of the present approach, as all local details of the neck geometry are contained in the single term $h_{\varepsilon }$. Note that in the limit as h→∞ we have C≈2/π and q≈4exp⁡(−2−πh) to obtain a specific (one dominant balance scaling) result for extremely thick-walled resonators. This is discussed in further detail in Part II.

### (d) Leading and first-order systems

The asymptotic form for ${\overset{ˇ}{h}}_{\varepsilon }$ both within a dipolar truncation and in the vanishing b limit, is easily obtained after introducing ${\overset{ˇ}{h}}_{\varepsilon }=4\text{i}{\overset{ˇ}{f}}_{\varepsilon }/(\pi b^{2})$ where

6.9 $$\begin{array}{rl} \lim_{b\to 0}{\overset{ˇ}{f}}_{\varepsilon } & \;=\left[\frac{1}{2}\left(1+\frac{b^{2}}{a^{2}}\right)-\frac{b^{2}}{8}+b^{2}\log\left(\frac{\varepsilon \sqrt{q}C(q)}{\sqrt{ab}}\right)\right]∼\frac{1}{2}\left(1+\frac{b^{2}}{a^{2}}\right)+b^{2}\log\left(\frac{\theta_{\text{ap}}\sqrt{b}\sqrt{q}C(q)}{\sqrt{a}}\right), \end{array}$$

which is the analogue to the earlier thin-walled expression (4.20). Hence we obtain the same dispersion equations as before, i.e. the leading-order expression in (4.19) and the first-order correction expression in (4.25*a*), but with the replacement $f_{\varepsilon }\mapsto {\overset{ˇ}{f}}_{\varepsilon }$, that is, for thick-walled resonators the first-order dispersion equation is

6.10 $$k_{B}^{2}=\frac{\left(f+1)[b^{2}f(2f-1)+{\overset{ˇ}{f}}_{\varepsilon }(f+1)(2{\overset{ˇ}{f}}_{\varepsilon }f-2{\overset{ˇ}{f}}_{\varepsilon }-2f+1)\right]}{b^{2}f\cos(2[\theta_{0}-\theta_{B}])+b^{2}f^{2}+{\overset{ˇ}{f}}_{\varepsilon }(2{\overset{ˇ}{f}}_{\varepsilon }-1)(f^{2}-1)}.$$

For reference, the analogue to the lowest-order approximation for thin resonators (4.21) follows straightforwardly, and finally, the dipolar estimate for the cut-off frequency of the first band now takes the form

6.11 $$k_{\text{max}}=\frac{2}{\bar{a}}{\left[1-8\log\left(\frac{\theta_{\text{ap}}\sqrt{\bar{b}}\sqrt{q}C(q)}{\sqrt{\bar{a}}}\right)\right]}^{-1/2}.$$

### (e) Numerical results

In this section, we briefly examine the validity of the multipole-matched asymptotic eigenvalue problem (4.13) with the replacement $h_{\varepsilon }\mapsto {\overset{ˇ}{h}}_{\varepsilon }$ described in (6.8), as well as the new first-order approximation for the first band in (6.10).

**Figure 8.**
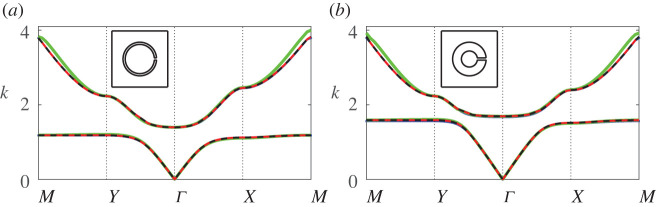
Band diagrams for a square array of thick-walled Helmholtz resonators for the relative wall thickness (or aspect ratio) h values: (*a*) h=1 and (*b*) h=4, with fundamental unit cells inset. Multipole results for dipole (green line), quadrupole (red line) and sextapole (dashed black line) truncations are superposed, in addition to finite-element results (blue line). In the above figures, we use $\bar{d}=1$, $\theta_{0}=0$, $\bar{b}=0.3$ and $\theta_{\mathrm{ap}}=\pi /48$. (Online version in colour.)

**Figure 9.**
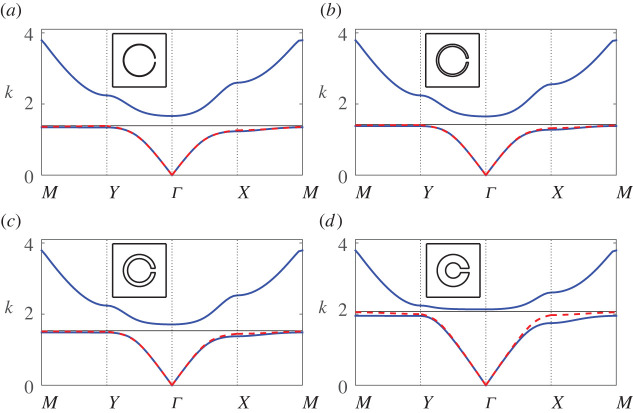
Band diagrams for a square array of thick-walled Helmholtz resonators as the wall thickness h is increased: (*a*) h=0.1, (*b*) h=0.5, (*c*) h=1 and (*d*) h=2, with fundamental unit cells inset. Blue lines denote results from finite-element methods, red dashed lines denote results from the first-order correction (6.10) and black lines denote estimates for the edge of the band gap (6.11). In the above figures, we use $\bar{d}=1$, $\theta_{0}=0$, $\bar{b}=0.3$ and $\theta_{\mathrm{ap}}=\pi /24$. (Online version in colour.)

In figure 8, we compare results from our updated eigenvalue formulation for various truncations (dipole L=1, quadrupole L=3 and sextapole L=5) against results obtained using finite-element methods, as we vary the thickness, or equivalently, the width of the resonator neck. We observe that for this narrow half-angle $\theta_{\mathrm{ap}}=\pi /48$, we achieve excellent agreement with finite-element benchmark results and rapid convergence, with results indistinguishable above dipole truncation and higher, for both bands. In figure 9 we examine the efficacy of the first-order approximation (6.8) for a slightly wider half-angle $\theta_{\mathrm{ap}}=\pi /24$, and observe very good agreement over a range of thickness h values; it becomes clear by figure 9*d* that for very large h the model is no longer able to accurately describe the first band towards the band edge, but that at longer wavelengths, the description is still accurate.

Reflecting upon figures 8 and 9, we observe that increasing h acts to close the band gap, to steepen the slope of the first band at lower frequencies and to translate the frequency range of the gap. This result is entirely consistent with the idea that as the thickness increases, the interior resonator shrinks so that the cut-off frequency increases.

**Figure 10.**
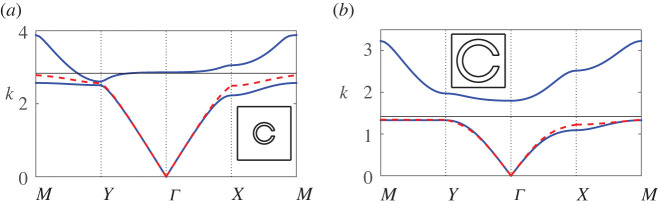
Band diagrams for a square array of thick-walled Helmholtz resonators as the outer radius b (equiv. filling fraction) is increased for fixed neck length ratio h=0.5: (*a*) b=0.2 and (*b*) b=0.4, with fundamental unit cells inset. Figure legends are identical to those in figure 9. In the above figures, we use $\bar{d}=1$, $\theta_{0}=0$ and $\theta_{\mathrm{ap}}=\pi /12$. (Online version in colour.)

Finally, we consider the relationship between resonator wall thickness and filling fraction in figure 10 where we impose a channel width aspect ratio of h=0.5 and vary the outer radius b for the aperture width $\theta_{\mathrm{ap}}=\pi /12$. We also superpose the first-order approximation for the first band (6.8), and the first band maximum (6.11). As in the thin-walled case, we observe a widening of the first band surface with increasing filling fraction (outer radius); this result may prove useful in countering the effect of thickness in the event that a wide band gap is sought. That is, although the presence of thickness may close the band gap it may be possible to compensate against this by tuning the outer radius. We find that the first band description generally works well, with the exception of configurations where the first and second band are almost degenerate at the Y point, and at this wider half-angle $\theta_{\mathrm{ap}}=\pi /12$ we observe a slight loss of accuracy in the band curvature near the saddle point at X. The behaviour of the first band gap closing at the Y high-symmetry point emphasizes the important point that the upper bound of the band gap cannot always be assessed from examining the spectrum at the Γ point alone.

## 7. Discussion

We have constructed a multipole formulation for calculating the band structure of a medium comprising a two-dimensional square array of thin- and thick-walled Helmholtz resonators embedded in a uniform fluid background. The eigenvalue problem was derived using both multipole methods and the method of matched asymptotic expansions, from which we were able to extract a dispersion equation approximation that implicitly defines the first band surface over the entire Brillouin zone. For thin-walled resonators we find that both the multipole formulation and the first-band surface description perform surprisingly well over a wide selection of aperture widths and filling fractions, compared with results from finite-element methods. Likewise, for thick-walled resonators, we find similarly strong performance across a selection of aperture widths and resonator neck thicknesses. A key feature of these Helmholtz resonator arrays is the emergence of a low-frequency band gap, where wave propagation through the array is not possible in the bulk material. We find that thin-walled resonators generally possess the widest gaps, and therefore for soundproofing applications recommend making the resonator walls as thin as practicably possible. The formulation we present also makes it possible to conveniently determine configurations that return a desired phase and/or group velocity at long wavelengths, should this be required. We anticipate that our multipole-matched asymptotic formulation will prove useful beyond the field of acoustics, such as in electromagnetism, after a simple replacement of constants (i.e. $B\mapsto \varepsilon_{r}^{-1}$ and $\rho \mapsto \mu_{r}$ [4]). The multipole-matched asymptotic expansion treatment outlined here provides closed-form expressions for the dispersion relation over a wide frequency range, which is particularly valuable, since it may be used to rapidly search over large parameter spaces for optimal configurations. Finally, we emphasize that the first band descriptions we obtain extend outside the classical long wavelength regime, and are therefore useful for describing how plane waves propagate through the array over very wide frequency ranges.

## Data Availability

This article has no additional data.

## Appendix A. Convergent lattice sum definition

The lattice sums $S_{\ell }^{Y}$ are most often defined via the conditionally convergent form [4]

A 1 $$S_{\ell }^{Y}({\text{k}}_{B})=\sum_{m,n}^{\;}{\;}^{\prime }Y_{\ell }(R_{mn}){\text{e}}^{\text{i}\ell \phi_{mn}}{\text{e}}^{\text{i}{\text{k}}_{B}\cdot {\text{R}}_{mn}},$$

where ${\text{R}}_{mn}=R_{mn}\exp(\text{i}\phi_{mn})$ is the (dimensionless) lattice generator in polar coordinates (i.e. $R_{mn}=(dm,dn)$ for a square lattice of period d and where m,n∈Z), ${\text{k}}_{B}=(k_{Bx},k_{By})$ is the dimensionless Bloch vector, and prime notation (with the sum) denotes summation over all points in the array excluding m=n=0. There are many ways in which this conditionally convergent sum may be regularized to obtain an absolutely convergent form [25]; we present the well-known expression for a square lattice as [4, eqn (3.104)]

A 2 $$S_{m}^{Y}({\text{k}}_{B})=\frac{1}{J_{t}(\xi )}\left[-\left[Y_{r}(\xi )+\frac{1}{\pi }\sum_{n=1}^{r}\frac{(r-n)!}{(n-1)!}{\left(\frac{2}{\xi }\right)}^{s}\right]\delta_{m,0}-\frac{4{\text{i}}^{m}}{d^{2}}\sum_{p,q}\frac{{\text{e}}^{\text{i}m\theta_{pq}}}{{\left(Q_{pq}\right)}^{r}}\frac{J_{t}(Q_{pq}\xi )}{Q_{pq}^{2}-1}\right],$$

where we define the regularization parameters r, denoting a small non-negative integer (e.g. r=3), and ξ, a small positive number which formally limits to zero (e.g. ξ=d/100). We also define the reciprocal lattice generator for a square lattice $K_{pq}=(2\pi p/d,2\pi q/d)$ where p,q∈Z, the translated generator ${\text{Q}}_{pq}=K_{pq}+{\text{k}}_{B}=Q_{pq}\exp(\text{i}\theta_{pq})$, along with s=r−2n+2 and t=m+r.
